# Quorum Sensing Promotes Phage Infection in Pseudomonas aeruginosa PAO1

**DOI:** 10.1128/mbio.03174-21

**Published:** 2022-01-18

**Authors:** Guanhua Xuan, Hong Lin, Lin Tan, Gang Zhao, Jingxue Wang

**Affiliations:** a Food Safety Laboratory, College of Food Science and Engineering, Ocean University of China, Qingdao, China; University of Nebraska-Lincoln

**Keywords:** *P. aeruginosa*, *las* quorum sensing, phage, adsorption, *galU*, lipopolysaccharide

## Abstract

Quorum sensing (QS) is used to coordinate social behaviors, such as virulence and biofilm formation, across bacterial populations. However, the role of QS in regulating phage-bacterium interactions remains unclear. Preventing phage recognition and adsorption are the first steps of bacterial defense against phages; however, both phage recognition and adsorption are a prerequisite for the successful application of phage therapy. In the present study, we report that QS upregulated the expression of phage receptors, thus increasing phage adsorption and infection rates in Pseudomonas aeruginosa. In P. aeruginosa PAO1, we found that *las* QS, instead of *rhl* QS, upregulated the expression of *galU* for lipopolysaccharide synthesis. Lipopolysaccharides act as the receptor of the phage vB_Pae_QDWS. This *las* QS-mediated phage susceptibility is a dynamic process, depending on host cell density. Our data suggest that inhibiting QS may reduce the therapeutic efficacy of phages.

## INTRODUCTION

Bacteriophage (phage) therapy has been suggested as an alternative to conventional antibiotic treatment in clinical practice (1). However, a successful phage therapy requires that we first overcome the wide variety of antiphage defense strategies that are present in bacterial hosts, including the CRISPR-Cas system, abortive infection systems, and prevention of phage adsorption (2, 3). Although the mechanisms of phage resistance have been widely studied, little is known about phage-host dynamics in the context of the microbial community. Quorum sensing (QS) is widely used by bacteria to coordinate group behavior, and it depends on the production and release of signal molecules termed “autoinducers” (AIs) (4, 5).

Phage adsorption is the first step by which phages recognize and bind to the bacterial cell surface (6). Recently, QS has been found to be involved in the antiphage process by reducing the number of phage receptors. Vibrio anguillarum exhibits downregulation of phage receptor OmpK expression in response to *N*-acyl-l-homoserine lactones (AHL), a class of QS-signaling molecules used by many Gram-negative bacteria (7). Vibrio cholerae modulates its sensitivity to phage infection via a mechanism that downregulates the phage receptor (lipopolysaccharide [LPS] O-antigen) and upregulates the expression of the hemagglutinin protease HAP when supplemented with the autoinducers CAI-1 or AI-2 (8). Both the aforementioned studies were based on the assumption that QS negatively regulates the expression of receptors required for phage infection. Phages can also communicate via the QS-like “arbitrium” system to alter infection outcomes (9, 10). V. cholera carrying the QS receptor VqmA expresses the autoinducer 3,5-dimethylpyrazin-2-ol (DPO), which acts as a cue for prophage induction when host cell densities are high (10). However, all QS-regulated phage resistance models have been developed only in *Vibrio* spp., and even though QS has been observed in several bacterial species, it is unknown whether QS plays a role in phage infection by modulating phage adsorption in other bacterial species.

Pseudomonas aeruginosa is a Gram-negative opportunistic pathogen which is responsible for the morbidity and mortality of patients with cystic fibrosis (11). Several QS systems have been described in P. aeruginosa, including the *las* and *rhl* systems, which recognize AHL signals (12, 13). In the *las* system, LasI synthesizes the signaling molecule *N*-(3-oxododecanoyl)-l-homoserine lactone (3O-C_12_-HSL). LasR binds to 3O-C_12_-HSL and functions as a transcriptional activator. In the *rhl* system, RhlI synthesizes C4-homoserine lactone (C4-HSL), which, in conjunction with RhlR, activates the expression of a second set of QS-related genes. The *las* system positively regulates the *rhl* system. Approximately 6% of P. aeruginosa genes are regulated by AHL-based QS systems (14, 15). Therefore, we speculated that certain receptors for P. aeruginosa phages may also be regulated by QS.

In the present study, we isolated and characterized Pseudomonas phage vB_Pae_QDWS, which could recognize and absorb LPS of P. aeruginosa. We identified a QS-regulated phage infection mechanism in P. aeruginosa PAO1, which is a model organism for the genus Pseudomonas. Our data showed that the expression of *galU*, which is a key gene for LPS synthesis, was upregulated by *las* QS, resulting in an increase in phage adsorption rate and subsequently increasing phage infection. In contrast to the results presented by previous studies, our study suggests that inhibiting QS may reduce the therapeutic efficacy of phage systems; this finding may help in filling several gaps in the field.

## RESULTS

### Phage genome analysis.

**FIG 1:**
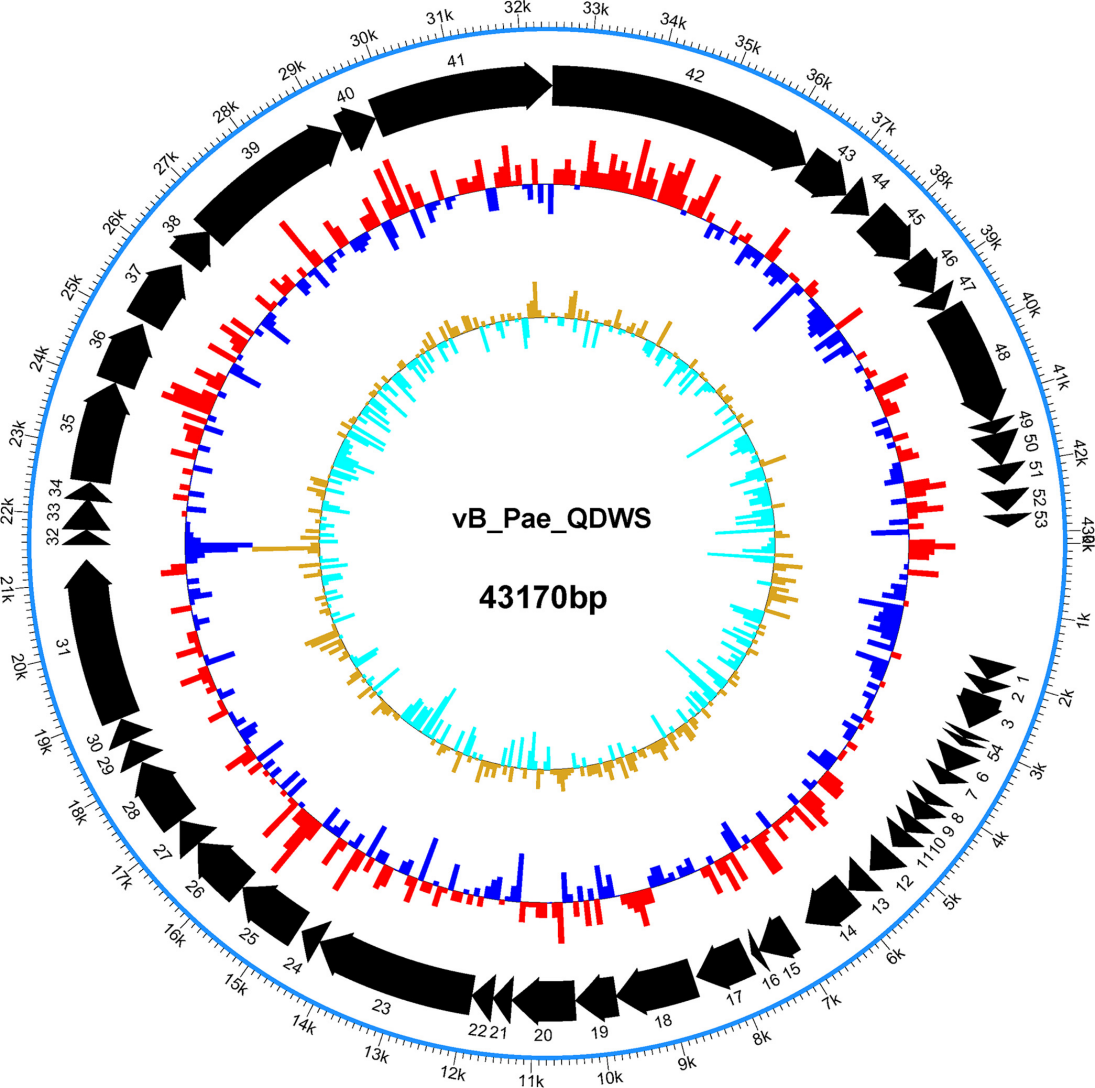
Genome organization of phage vB_Pae_QDWS. The first circles represent the 53 open reading frames (ORFs) on the sense strand of the phage. The second circle shows G+C content. The red outward and blue inward arrows indicate that the G+C content of that region is higher or lower than the average G+C content of the whole genome, respectively. The third circle shows the G+C skew.

The genome of phage vB_Pae_QDWS is a 43,170-bp, double-stranded DNA molecule with 62.3% G+C content and contains 53 coding DNA sequences (CDSs), which are transcribed in the same direction (Fig. 1). Bioinformatics analysis revealed 21 gene products with known functions, and the remaining 32 genes were presumed to encode hypothetical proteins. An overview of the functional prediction of phage-encoded gene products is provided in Table 1. No genes related to phage lysogeny were identified, confirming the lytic characteristics of phage vB_Pae_QDWS. Compared to other phage genome sequences obtained from the NCBI GenBank repository, the genome sequence of phage vB_Pae_QDWS most closely resembled that of the P. aeruginosa phage phiKMV (16, 17), with a similarity of 94% and a coverage of 92%. The complete genome sequence of phage vB_Pae_QDWS has been deposited in GenBank under the accession number MZ687409.

**TABLE 1:** Functional genes of bacteriophage vB_Pae_QDWS

ORF no.	Function	Sequence length (aa)
14	DNA-binding protein	269
15	DNA primase	180
18	DNA_B helicase	397
20	DNA ligase	315
23	DNA polymerase	807
27	Endonuclease VII	146
31	RNA polymerase	815
35	Head-tail connector protein	510
36	Capsid and scaffold protein	322
37	Capsid protein	335
38	Tail tubular protein A	184
39	Tail tubular protein B	835
41	Internal virion protein	898
42	Internal virion protein	1,337
43	Particle protein	251
45	Structural protein	288
46	Tail fiber protein	201
48	Terminase large subunit	601
49	Holin	66
50	Endolysin	160
52	Minor structural protein	104
Others	Hypothetical protein	

**FIG 2:**
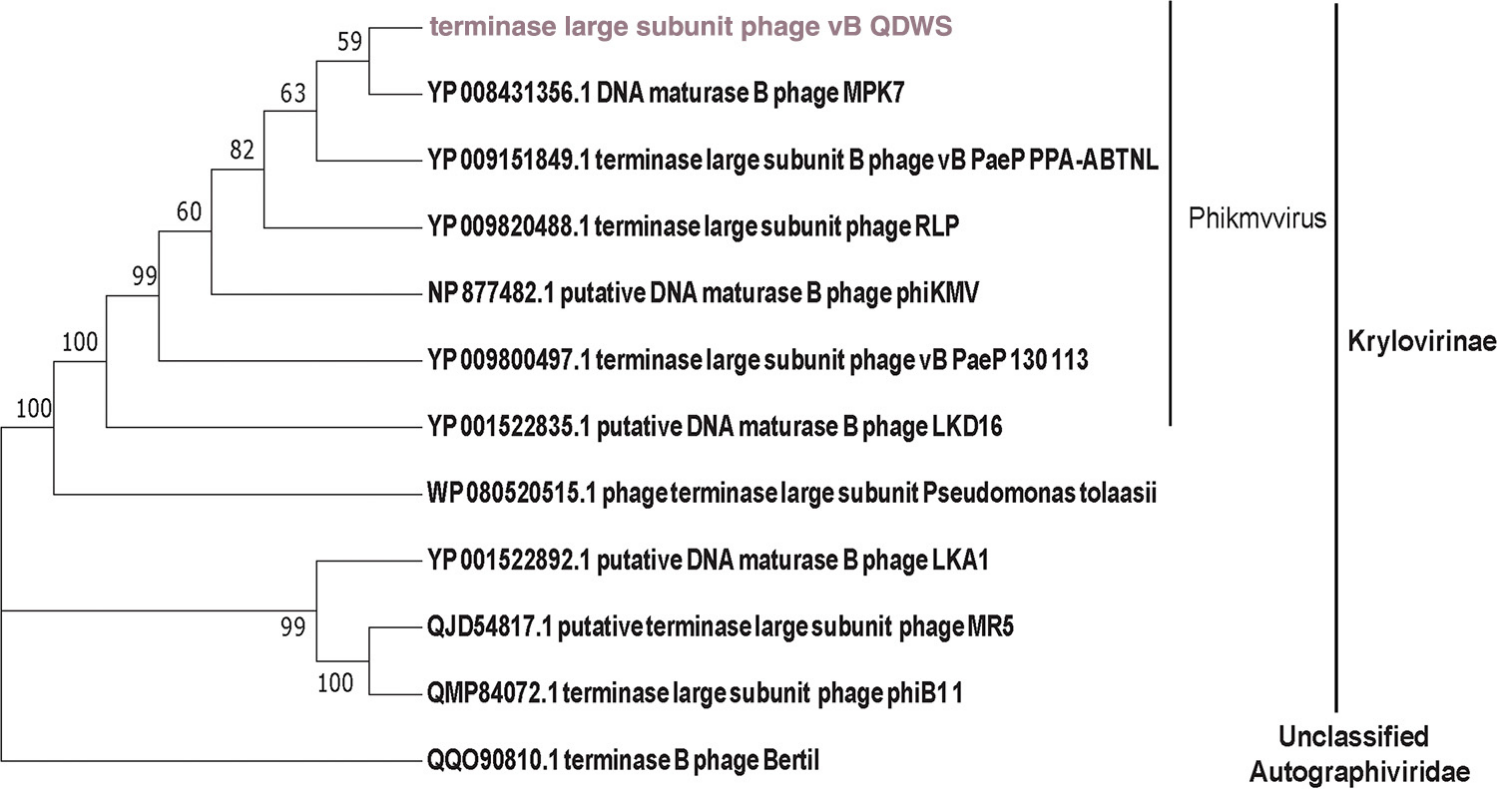
Phylogenetic tree based on amino acid sequences of terminase large subunit protein of phage vB_Pae_QDWS and related phages.

One-step growth curve analysis revealed that phage vB_Pae_QDWS had a latency period of approximately 10 min (Fig. S1 in the supplemental material). The final titers of phage exceeded 10^11^ PFU/mL, indicating that they were highly infective toward P. aeruginosa PAO1. Phylogenetic analysis based on the amino acid sequence of the large subunit of the terminase protein from each phage showed that P. aeruginosa phage vB_Pae_QDWS was most closely related to *Phikmvvirus*, which belongs to subfamily *Krylovirinae* and family *Autographiviridae* (Fig. 2).

10.1128/mBio.03174-21.1 FIG S1 One-step growth curve of phage vB_Pae_QDWS on P. aeruginosa strain PAO1. P. aeruginosa PAO1 was grown in LB medium until the log-phase (OD_600_, ∼0.4 to 0.6). Phage vB_Pae_QDWS was then added to the PAO1 culture at a multiplicity of infection (MOI) of 0.01 and incubated at 37°C. Using the double-layer-agar plate method, we determined the free bacteriophage count at each time point. All data are averages of six samples with standard deviations (error bars). Download FIG S1, TIF file, 0.8 MB. Copyright © 2022 Xuan et al. 2022 Xuan et al. https://creativecommons.org/licenses/by/4.0/ This content is distributed under the terms of the Creative Commons Attribution 4.0 International license.

### *las* QS influences phage resistance.

**FIG 3:**
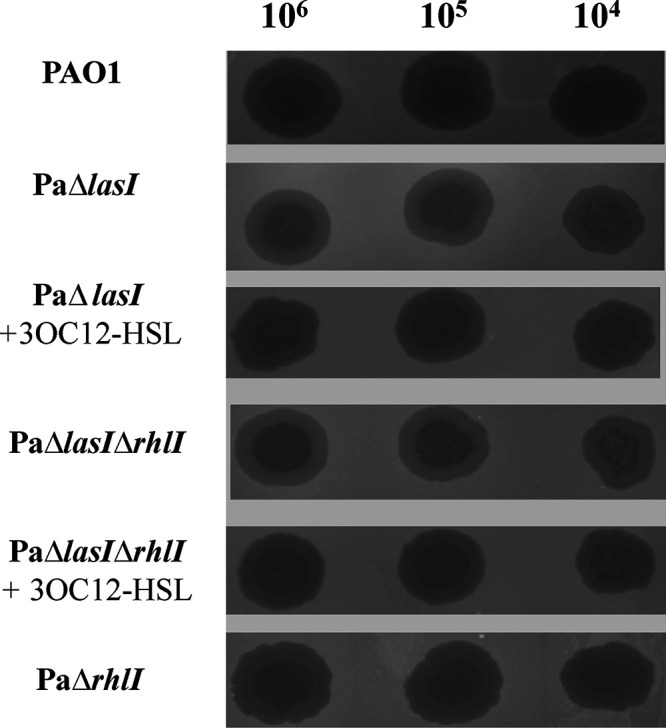
Phage sensitivity assay. Tenfold serial dilutions of phage vB_Pae_QDWS plated on wild-type Pseudomonas aeruginosa PAO1 and quorum-sensing (QS) mutants PaΔ*lasI*, PaΔ*rhlI*, and PaΔ*lasI*Δ*rhlI*. Ten-micromolar 3O-C_12_-HSL was added to examine its effect on phage sensitivity.

We investigated the effects of the *las* and *rhl* QS systems on phage vB_Pae_QDWS resistance. Deletion of *lasI* increased the resistance of P. aeruginosa PAO1 to phage infection. However, the deletion of *rhlI* did not affect the transparency of the plaques, suggesting that this deletion did not affect the resistance of the bacteria to this phage (Fig. 3). When exogenous 3O-C_12_-HSL was added, both PaΔ*lasI* and PaΔ*lasI*Δ*rhlI* restored sensitivity toward phage vB_Pae_QDWS (Fig. 3). The plaques of the complemented strain Δ*lasI*::*lasI* were more transparent than that of the strain PaΔ*lasI* (Fig. S2). These results suggest that the *las* QS system, but not the *rhlI* QS system, positively regulates phage sensitivity of P. aeruginosa PAO1.

10.1128/mBio.03174-21.2 FIG S2 Phage sensitivity assay. Tenfold serial dilutions of phage vB_Pae_QDWS were plated on Pseudomonas aeruginosa PAO1 (pBBR5), PaΔ*lasI* (pBBR5), and PaΔ*lasI::lasI*. Download FIG S2, TIF file, 1.5 MB. Copyright © 2022 Xuan et al. 2022 Xuan et al. https://creativecommons.org/licenses/by/4.0/ This content is distributed under the terms of the Creative Commons Attribution 4.0 International license.

**FIG 4:**
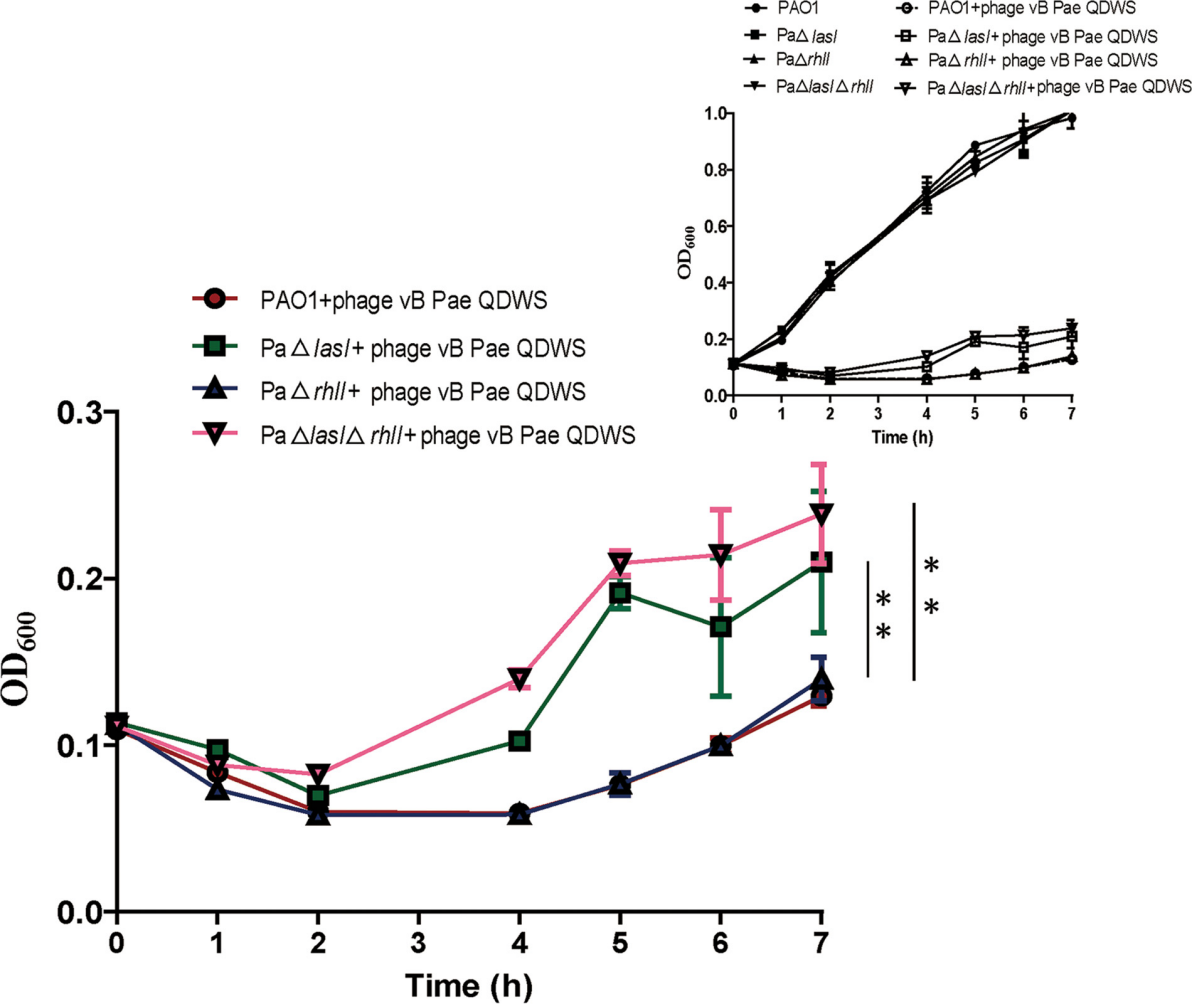
Growth curves of Pseudomonas aeruginosa PAO1 strains in LB medium. Optical densities (OD_600_) of cultures of PAO1 wild-type (WT) and QS mutants PaΔ*lasI*, PaΔ*rhlI*, and PaΔ*lasI*Δ*rhlI* in the presence or absence of phage vB_Pae_QDWS at a multiplicity of infection (MOI) of 0.1 were measured in a 96-well microtiter plate containing 200 μL of each culture using a Synergy H1 microplate reader at different incubation times. Data are averages of six samples with standard deviations (error bars). **, *P < *0.01 (two-way analysis of variance [ANOVA]).

Phage vB_Pae_QDWS reduced cell density in the cultures of wild-type PAO1 and QS mutants PaΔ*lasI*, PaΔ*rhlI*, and PaΔ*lasI*Δ*rhlI* compared to that in control cultures without the phage. However, PaΔ*lasI* and PaΔ*lasI*Δ*rhlI* exhibited a slower reduction in cell density within 2 h and a more rapid regrowth of cells during the remainder of the incubation period than PAO1 and PaΔ*rhlI* (Fig. 4).

### *las* QS affects phage adsorption.

**FIG 5:**
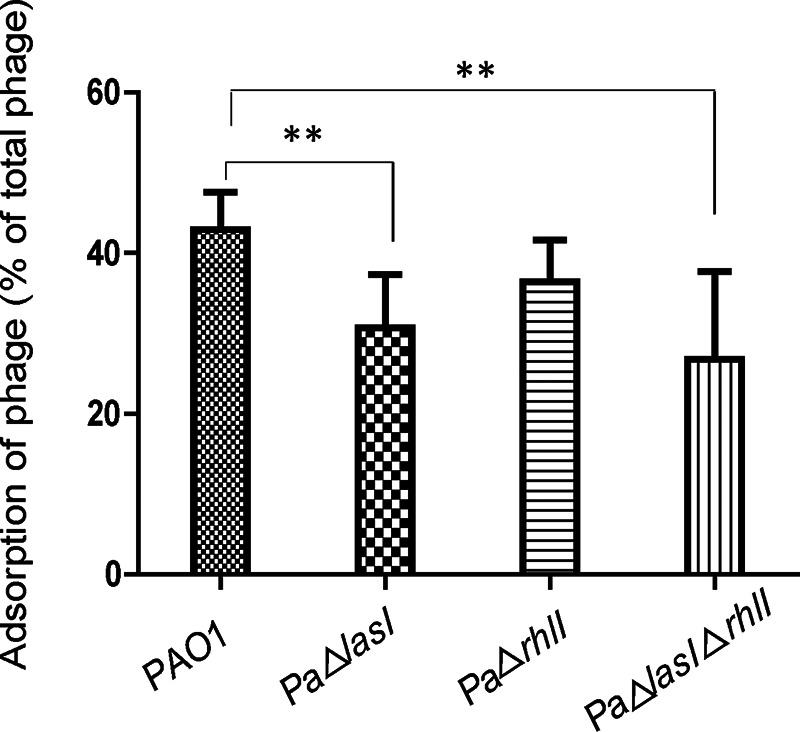
Adsorption rate of phage vB_Pae_QDWS by its host strain Pseudomonas aeruginosa PAO1 wild-type (WT) and quorum-sensing (QS) mutants. Data are averages of six samples with standard deviations (error bars). **, *P < *0.01 (Student’s paired *t* test).

To investigate the mechanisms associated with the altered susceptibility of P. aeruginosa PAO1 strains to phage infections, the adsorption rate of phage vB_Pae_QDWS by different P. aeruginosa PAO1 strains was examined. The single Δ*lasI* and double Δ*lasI*Δ*rhlI* mutants exhibited pronounced reduction in phage adsorption rates compared to that by the wild-type strain. The single Δ*rhlI* mutant, however, exhibited no differences in adsorption rate compared to that of the wild-type strain (Fig. 5). Thus, *las* QS positively regulated phage susceptibility by increasing the phage adsorption rate.

**FIG 6:**
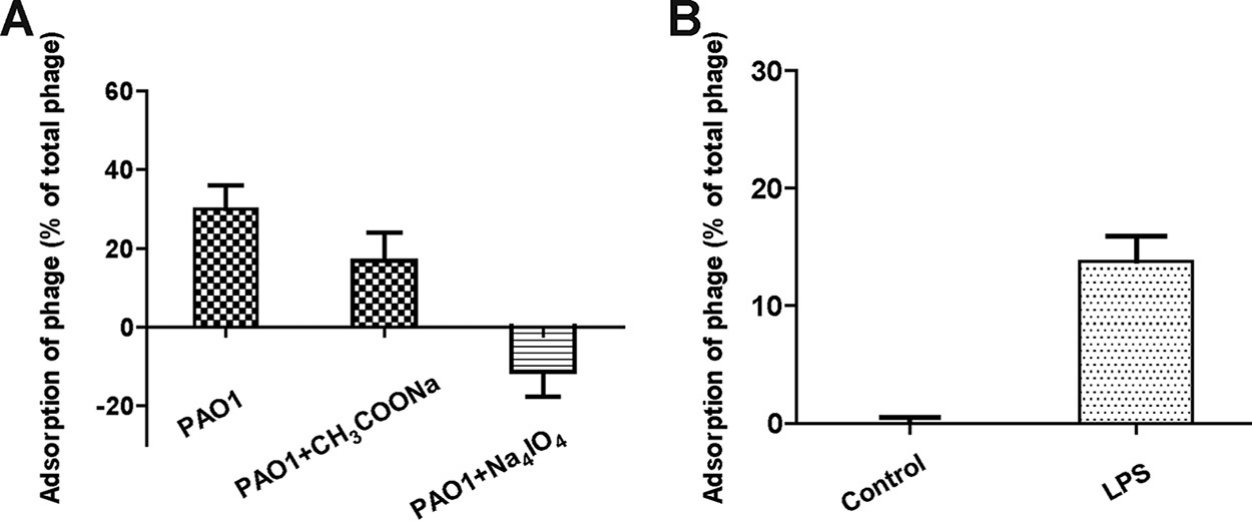
Identification of lipopolysaccharide (LPS) as an important receptor for Pseudomonas phage vB_Pae_QDWS infection. (A) Na_4_IO_4_ treatment significantly reduced the adsorption of Pseudomonas phage vB_Pae_QDWS. (B) Extracted LPS was used for adsorption assays. The adsorption rate was increased in the LPS-treated group compared to that in the control group. Data are averages of three samples with standard deviations (error bars).

Na_4_IO_4_ was used to treat P. aeruginosa cells and damage LPS. Adsorption assay results showed that Na_4_IO_4_ treatment led to a dramatic reduction in the adsorption rates. In contrast, sodium acetate (CH_3_COONa) treatment resulted in a modest reduction in the adsorption rate, which may be due to the toxic effects of the solvent (Fig. 6A). The extracted LPS was used for adsorption assays. There was a significant increase in the adsorption rate when LPS was added to the reaction system (Fig. 6B). Thus, LPS was recognized as a receptor for Pseudomonas phage vB_Pae_QDWS.

### *GalU* expression is activated by *las* QS.

**FIG 7:**
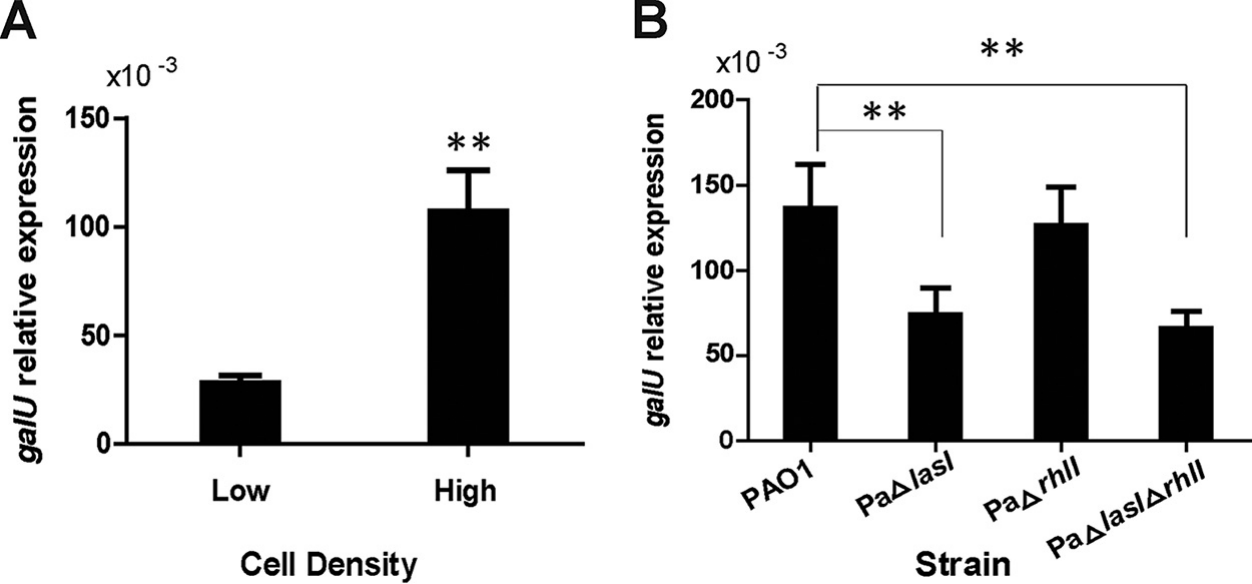
*las* QS activates *galU* expression. (A) Relative *galU* expression measured by RT-qPCR in Pseudomonas aeruginosa PAO1 cells at low and high cell densities (OD_600_, 0.8 and 2.5, respectively). The reference gene was *rplS*. (B) Relative *galU* expression at high cell density in wild-type (WT) PAO1 and the designated QS mutants. Data are averages of three samples with standard deviations (error bars). **, *P < *0.01 (paired *t* test).

*GalU* is involved in P. aeruginosa LPS core synthesis (18, 19). The expression of *galU* is dependent on the growth phase, with its expression at high cell densities being higher than that at low cell densities (Fig. 7A). High cell density should lead to higher phage susceptibility due to the synthesis of more LPS receptors. As expected, the adsorption rate of stationary-phase cells was significantly higher than that of logarithmic-phase cells, and the optical density of stationary-phase cells decreased faster than that of the logarithmic-phase cells (Fig. S3). We also investigated *galU* expression in different P. aeruginosa PAO1 strains. When *lasI* was deleted, the expression level of *galU* was significantly decreased. However, *galU* expression did not change in strain PaΔ*rhlI* compared to that in the wild-type strain (Fig. 7B). Hence, we concluded that *galU* expression is regulated by *las* QS.

10.1128/mBio.03174-21.3 FIG S3 Cells at different growth phases exhibited different susceptibility to phage infection. Overnight cultures were inoculated into LB medium and sampled at logarithmic phase (OD_600_, 0.8) and stationary phase (OD_600_, 3) for determination of adsorption rate (A) and phage lysis kinetics (B). The lysis kinetics for phage vB_Pae_QDWS was at an MOI of 0.1 on Pseudomonas aeruginosa strain PAO1 and was detected in a 96-well microtiter plate containing 200-μL cultures using a Synergy H1 microplate reader. All data are averages from six samples with standard deviation (error bar). The experiment was repeated at least three times. **, *P* < 0.01 (paired *t* test). Download FIG S3, TIF file, 0.8 MB. Copyright © 2022 Xuan et al. 2022 Xuan et al. https://creativecommons.org/licenses/by/4.0/ This content is distributed under the terms of the Creative Commons Attribution 4.0 International license.

## DISCUSSION

**FIG 8:**
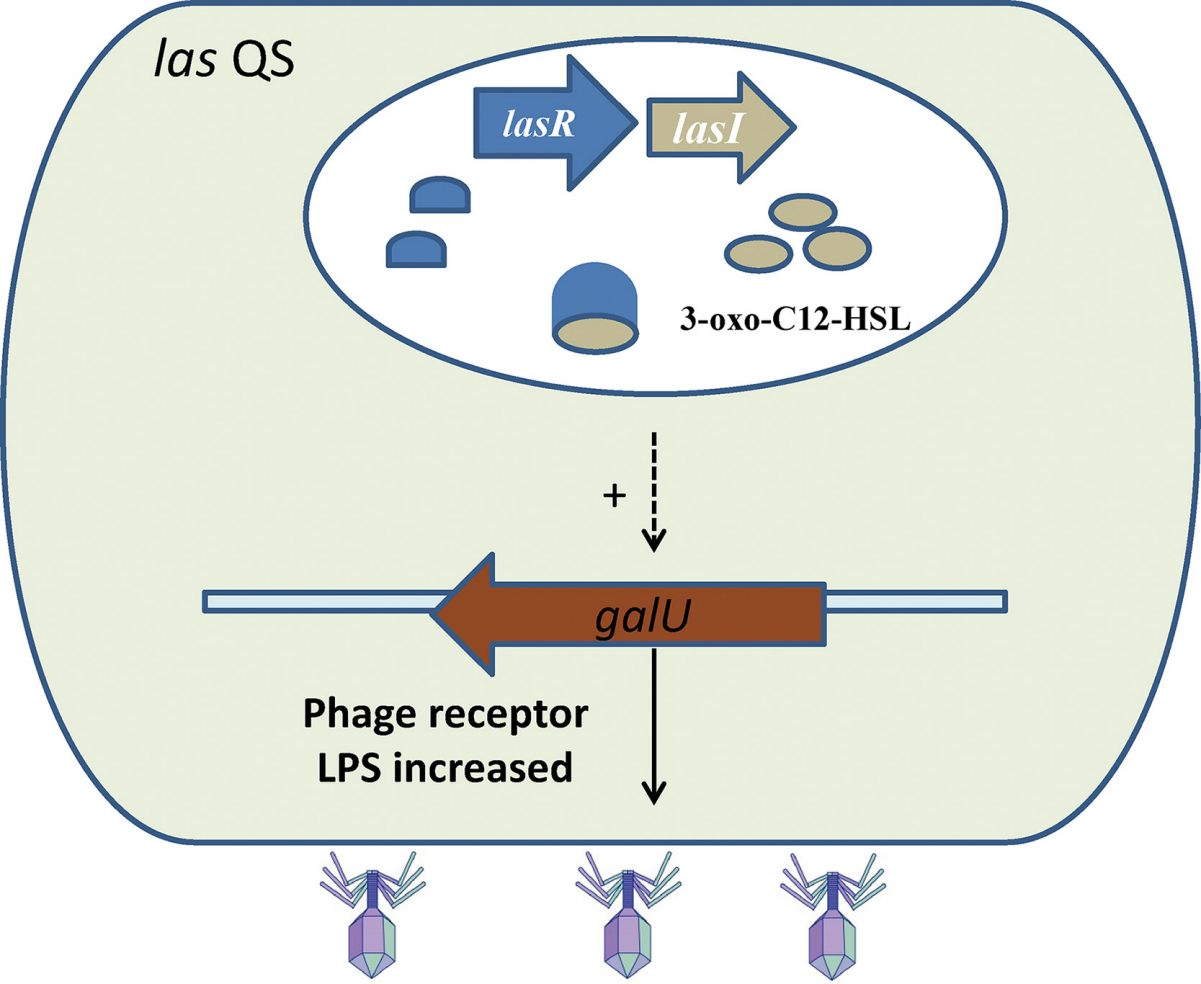
Schematic representation of the mechanism by which *las* QS regulates the resistance of Pseudomonas aeruginosa PAO1 to phage vB_Pae_QDWS. The *las* QS positively regulates the expression of *galU*, which is involved in LPS biosynthesis, thereby promoting phage adsorption.

Taken together, our findings indicate that *las* QS regulates *galU* expression, which is essential for LPS receptor synthesis and subsequently affects the susceptibility of P. aeruginosa PAO1 to phage vB_Pae_QDWS infection. A schematic of the proposed mechanism is shown in Fig. 8. Disruption of *las* QS led to an increase in bacterial resistance to phage infection; however, this resistance decreased after the addition of synthetic 3O-C_12_-HSL (Fig. 3). These results were further supported by the results of growth and adsorption assays of P. aeruginosa PAO1 and its QS mutants (Fig. 4 and 5). Cells at high density express more receptors and are more susceptible to phage infection than cells at low cell density. Thus, our results suggest that QS positively regulates phage susceptibility in PAO1 cells.

The expression of CRISPR-Cas is regulated by QS. Pseudomonas aeruginosa strain PA14 and *Serratia* use QS to activate *cas* gene expression, which protects the bacteria against phage infection (20, 21). In contrast, in the present study, we showed that PAO1 QS could improve the efficacy of phage therapy. Broniewski reported that inhibiting QS may reduce the therapeutic efficacy of phages (15), which is consistent with our results. It is likely that QS plays a dual role by decreasing phage adsorption rates and favoring the evolution of CRISPR immunity in P. aeruginosa. Since PAO1 does not possess the CRISPR-Cas system, whereas PA14 does (22), QS may have different effects upon phage infection in both strains. Cells of strain PAO1 are easily lysed by phages that use LPS as a receptor, under high cell density. We showed that P. aeruginosa PAO1 QS increased phage adsorption, which is different from previous observations in V. anguillarum, V. cholerae, and Escherichia coli (10, 23, 24). However, type IV pili are recognized by many Pseudomonas phages and are positively regulated by QS, which is in agreement with our results (15, 25, 26). The regulation of phage resistance by QS is clearly diverse and complex.

The genes *wzy*, *wbpD*, *galU*, and *wzz* are closely related to LPS synthesis (18, 19, 27, 28). In the present study, transcriptional analysis revealed that *galU* expression was closely related to *las* QS (Fig. 7), but the expression of *wzy*, *wbpD*, and *wzz* was not related to *las* QS (Fig. S4). Furthermore, the expression of *galU* was elevated at high cell density and regulated by *las* QS. However, *rhl* QS had no effect on *galU* expression or the susceptibility of the bacterial strain to phage vB_Pae_QDWS (Fig. 3). Transcriptional data also showed that *galU* was not controlled by *rhl* QS (Fig. 7B). The *las* system exerts positive control over the *rhl* system in P. aeruginosa (29). In some cases, the two systems have opposing effects on the same target. There are many genes that are specifically regulated by either the *las* or the *rhl* system (30, 31). The *las* and *rhl* QS systems regulate 315 genes, while the *rhl* system regulates approximately 112 genes (32, 33). It is, therefore, expected that phage vB_Pae_QDWS infection efficiency is controlled by *las* QS rather than by *rhl* QS.

10.1128/mBio.03174-21.4 FIG S4 Transcriptional analysis of *wzy*, *wbpD,* and *wzz* in Pseudomonas aeruginosa PAO1 and the designated QS mutants. Total RNA was extracted from cells at an OD_600_ of 2.5. Relative gene expression normalized to *rplS* expression was measured by RT-qPCR. Data are averages of three samples with standard deviations (error bars). Download FIG S4, TIF file, 0.9 MB. Copyright © 2022 Xuan et al. 2022 Xuan et al. https://creativecommons.org/licenses/by/4.0/ This content is distributed under the terms of the Creative Commons Attribution 4.0 International license.

QS-mediated phage infection is a dynamic process. *las* QS is usually affected by different growth conditions (31, 34) and bacterial community composition (35–37), which may, in turn, affect phage resistance. In the present study, we found that phage resistance also depends on the growth phase of the host. Stationary-phase PAO1 cells were more susceptible to infection than logarithmic-phase cells (Fig. S3). This discovery will be significant for guiding the preparation of high-titer phage vB_Pae_QDWS because there is no optimal, universal method for phage amplification (38).

Our findings represent an example of evolutionary trade-offs. P. aeruginosa relies on QS to regulate several functions, including the expression of virulence factors and biofilm development (11, 39). The virulence factor LPS acts as a phage receptor that is conducive to phage infection, and its synthesis pathway is positively regulated by QS (Fig. 7) (40). QS has the potential to mediate trade-offs between LPS-based bacterial virulence and phage sensitivity. LPS is one of the factors involved in biofilm formation (41), which is positively regulated by QS (42), increases the resistance of microorganisms toward biocides, and reduces antibiotic treatment efficacy. Although the coevolutionary mechanisms involved in antibiotic resistance and phage sensitivity have been widely studied (43, 44), our discovery adds another example of pleiotropy involving antibiotic resistance and phage sensitivity driven by QS.

In summary, we discovered that *las* QS plays a significant role in regulating phage vB_Pae_QDWS susceptibility in PAO1. *GalU*, which contributes to LPS synthesis, is positively regulated by *las* QS. Since LPS is a common receptor for Pseudomonas phages, *las* QS-regulated phage killing is probably a conserved mechanism.

## MATERIALS AND METHODS

### Strains, plasmids, and growth conditions.

Detailed information of the strains and plasmids used in the present study is presented in Table S1 in the supplemental material. All PCR primers used in the study are listed in Table S2. P. aeruginosa was cultured in Luria-Bertani (LB) medium at 37°C. Gentamicin (30 μg/mL) and tetracycline (30 μg/mL) were added as required.

10.1128/mBio.03174-21.5 TABLE S1 Strains and plasmids used in the present study. Download Table S1, DOCX file, 0.03 MB. Copyright © 2022 Xuan et al. 2022 Xuan et al. https://creativecommons.org/licenses/by/4.0/ This content is distributed under the terms of the Creative Commons Attribution 4.0 International license.

10.1128/mBio.03174-21.6 TABLE S2 Primers used in the present study. Download Table S2, DOCX file, 0.02 MB. Copyright © 2022 Xuan et al. 2022 Xuan et al. https://creativecommons.org/licenses/by/4.0/ This content is distributed under the terms of the Creative Commons Attribution 4.0 International license.

### Isolation and purification of phages.

Phages specific for P. aeruginosa PAO1 were isolated from sewage samples collected in Qingdao, China. The sewage samples were centrifuged at 2,348 × *g* for 10 min and then filtered through a 0.22-μm-pore-size filter (Millipore, Burlington, MA, USA). The filtrate was mixed with 50 mL of log-phase P. aeruginosa PAO1 cells and incubated at 37°C with 200-rpm rotary agitation for 12 h. The resulting culture suspension was centrifuged and filtered, as described above. Phages were isolated using the double-layer agar plate method (45). Single plaques were separated by stinging with a pipette tip into the plaque followed by resuspending the phages in SM buffer (100 mM NaCl, 8 mM MgSO_4_, 50 mM Tris-HCl, pH 7.5). After multiple rounds of purification, the phage was verified by electron microscopy.

### Gene sequencing and bioinformatic analysis.

Genomic DNA of phage vB_Pae_QDWS was extracted using a bacterial DNA kit (Omega) according to the manufacturer’s instructions. DNA sequencing was performed by Shanghai Biozeron Biotechnology Co., Ltd. (Shanghai, China). Phage DNA library construction and genome sequencing were performed using the Illumina MiSeq sequencing platform to obtain paired-end reads. The genome sequence was assembled using ABySS (http://www.bcgsc.ca/platform/bioinfo/software/abyss). GapCloser software (https://sourceforge.net/projects/soapdenovo2/files/GapCloser/) was subsequently used to fill the remaining local internal gaps and correct single nucleotide polymorphisms (SNPs) for final assembly. Genome annotation was performed using the *ab initio* prediction method. Gene models were identified using GeneMark server (http://topaz.gatech.edu/GeneMark/genemarks.cgi). All gene models were evaluated by performing BLASTp searches using the nonredundant (nr) NCBI GenBank database, Swiss-Prot, KEGG, and COG to perform functional annotation.

The terminal enzyme large subunit sequence of phage vB_Pae_QDWS was used as a query to identify homologues in sequenced bacterial genomes at NCBI (http://blast.ncbi.nlm.nih.gov/). Eleven terminase large subunit protein sequences of different phages with high identity were selected, combined with the seed protein from phage vB_Pae_QDWS for phylogenetic tree analysis. Multiple-sequence alignment was carried out using ClustalW (46), and the tree was constructed by MEGA version 7.0 (47) using neighbor joining with a pairwise deletion, *p*-distance distribution, and bootstrap analysis of 1,000 repeats as the parameters.

### Gene knockout and complementation.

All deletions in P. aeruginosa PAO1 were performed according to a previously published method (48). The primers used for inactivation of Pa*lasI* and Pa*rhlI* are listed in Table S2. The mutants PaΔ*lasI*, PaΔ*rhlI*, and PaΔ*lasI*Δ*rhlI* were selected using colony PCR. The complemented strain was constructed by transforming pBBR-*lasI* with gentamicin resistance into PaΔ*lasI* to generate Δ*lasI*::*lasI*.

### Phage sensitivity assay.

Overnight cultures of P. aeruginosa PAO1, PaΔ*lasI*, PaΔ*rhlI*, and PaΔ*lasI*Δ*rhlI* strains were inoculated in fresh LB medium for 5 h until the early stationary phase (optical density at 600 nm [OD_600_], 2) was reached. Then, 100 μL of the culture was mixed with 5 mL of melted 1% agar and LB medium to prepare double-layered agar plates. For *N*-(3-oxododecanoyl)-l-homoserine lactone (3O-C_12_-HSL) chemical complementation experiments, 3O-C_12_-HSL was stored in dimethyl sulfoxide (DMSO) and added to the melted 1% agar and LB medium to form a double layer of agar at a final concentration of 10 μM. In control samples, an equivalent volume of DMSO was added as a solvent control. The phages were then subjected to 10-fold gradient dilution in SM buffer, and 3-μL aliquots were spotted onto a plate and incubated at 37°C for 12 h.

### Adsorption rate assay.

Overnight cultures (OD_600_, 0.05) of P. aeruginosa PAO1, PaΔ*lasI*, PaΔ*rhlI*, and PaΔ*lasI*Δ*rhlI* were inoculated in fresh LB medium. The cells were cultured until the OD_600_ reached 2.5, followed by 10-fold dilution in LB medium. To facilitate phage adsorption, 0.5 mL of phage solution (10^5^ PFU/mL) was mixed with the diluted cell suspension (0.5 mL) and incubated at 37°C for 5 min. LB broth mixed with phage without bacteria was used as the control. The cultures were then centrifuged at 7,378 × *g* for 2 min, and the titer of free phage in the supernatant was determined using the double-layer agar method (45). The phage adsorption rate was calculated as follows: adsorption rate (%) = [(initial phage titer − phage titer in the supernatant)/(initial phage titer)] × 100.

LPS was extracted using an LPS extraction kit (iNtron Biotechnology, China). The concentration of LPS was determined using the phenol-sulfuric acid method (49). For LPS adsorption assays, 40 μL of extracted LPS (0.8 mg/mL) was added to 0.5 mL LB broth and mixed with the phage (10^5^ PFU/mL) at 37°C for 20 min to allow adsorption. Control samples were transferred into LB broth with 40 μL of phosphate-buffered saline (PBS) before mixing with phages. Samples were centrifuged at 9,000 × *g* at 4°C for 10 min, and then their titers were determined.

### Identification of phage receptor.

Overnight cultures of the P. aeruginosa strains were diluted (1:100) in LB medium and incubated at 37°C until the OD_600_ reached 2. The cells were then treated with 50 mM Na_4_IO_4_ at 37°C for 30 min. A control cell suspension containing only solvent CH_3_COONa was prepared. The phage adsorption rate was determined as described above.

### RT-qPCR.

Cells were harvested at the indicated OD_600_. RNA was purified using the TRIzol RNA purification kit (catalog no. 12183555; Invitrogen). Total cDNA was synthesized using the HiScript II reverse transcriptase kit (Vazyme). Real-time quantitative reverse transcription-PCR (RT-qPCR) was performed using the SYBR green real-time PCR master mix and StepOnePlus real-time PCR system (ABI). To calculate the relative expression levels of the tested genes, *rplS* was used as the reference gene.

### Statistical analysis.

Data were expressed as means ± standard deviation, and differences between groups were evaluated using Student's *t* test for individual measurements (Fig. 5 and 7) or two-way analysis of variance (ANOVA) for data containing repeated measurements of the same cultures (Fig. 4). Analysis was carried out using GraphPad Prism v.5 software.

### Data availability.

The complete genome sequence of phage vB_Pae_QDWS has been deposited in GenBank under the accession number MZ687409.
