# Supplementary figures and images for: Quorum Sensing Promotes Phage Infection in Pseudomonas aeruginosa PAO1
Source: mBio. 2022 Jan 18;13(1):e03174-21. doi: 10.1128/mbio.03174-21 (PMC8764535; doi:10.1128/mbio.03174-21)

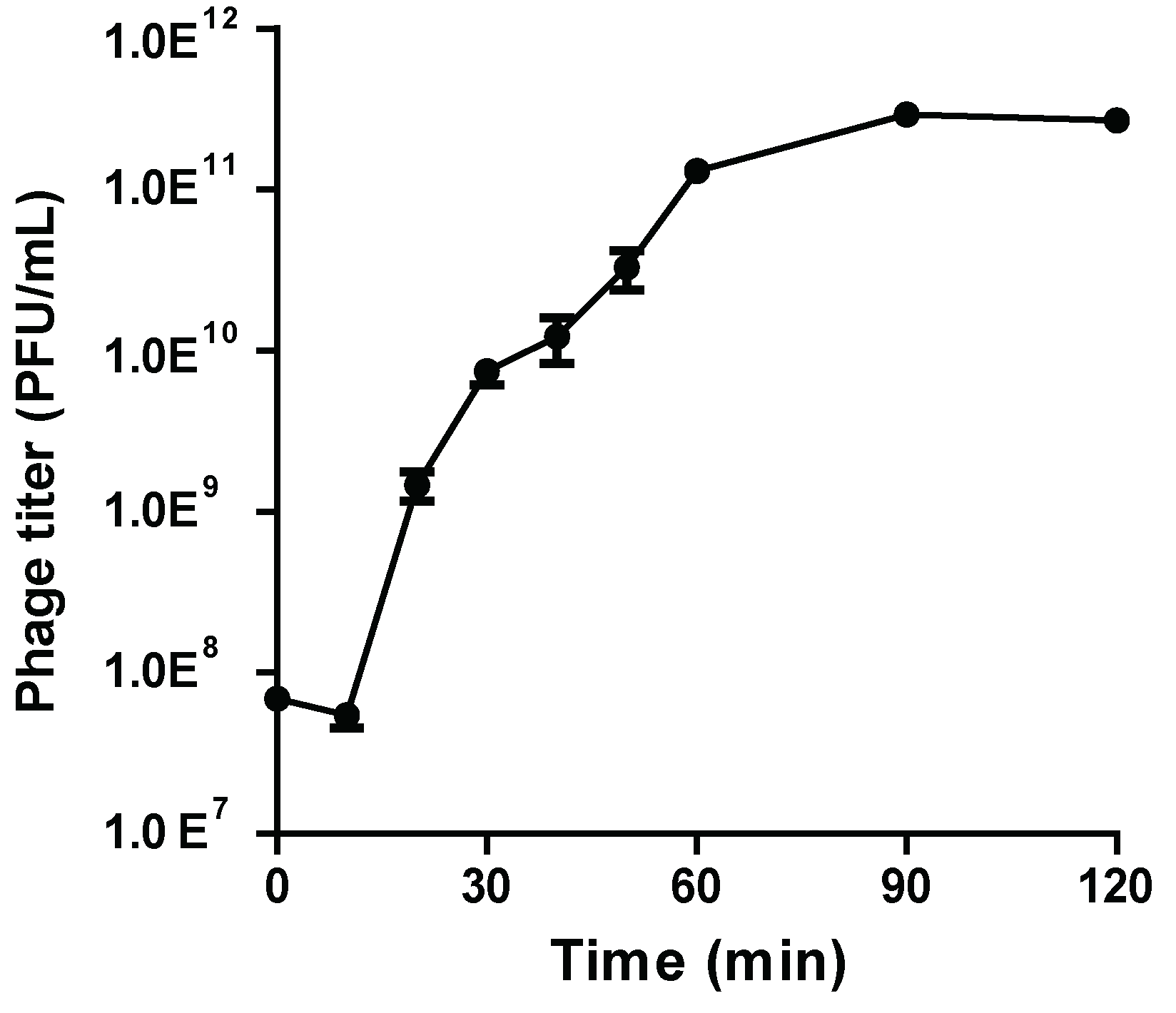

Supplement: FIG S1 [file mbio.03174-21-sf001.tif]

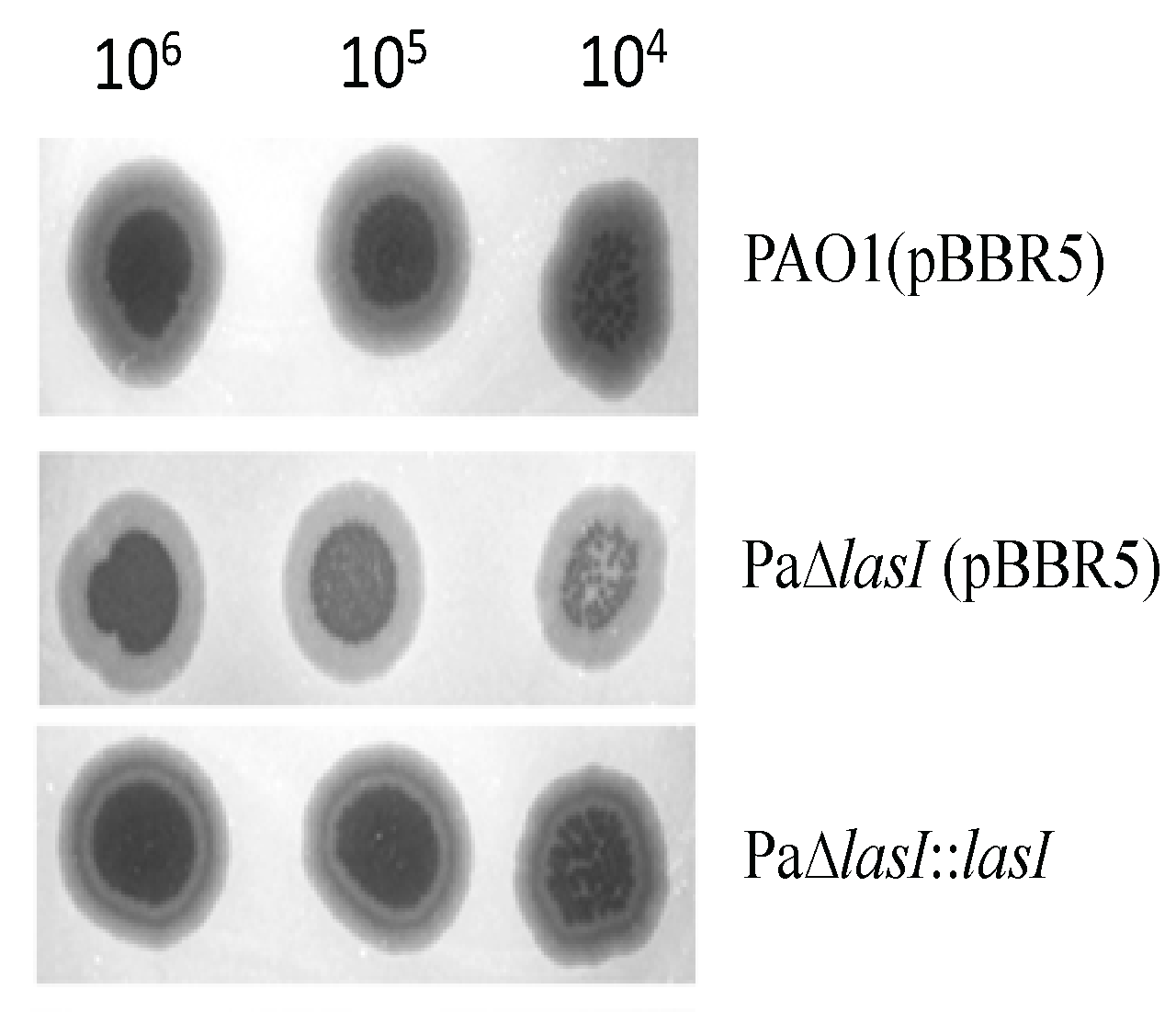

Supplement: FIG S2 [file mbio.03174-21-sf002.tif]

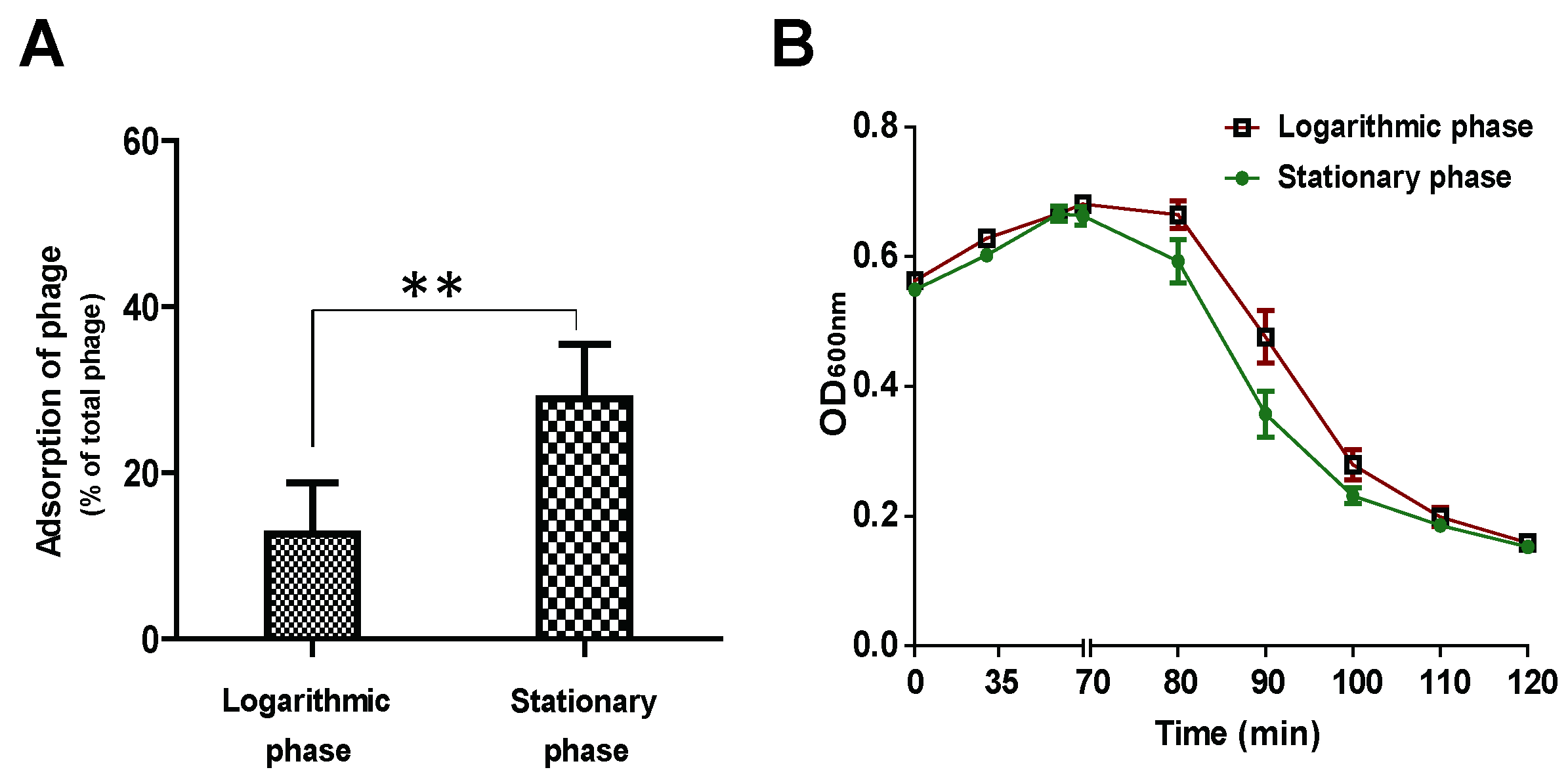

Supplement: FIG S3 [file mbio.03174-21-sf003.tif]

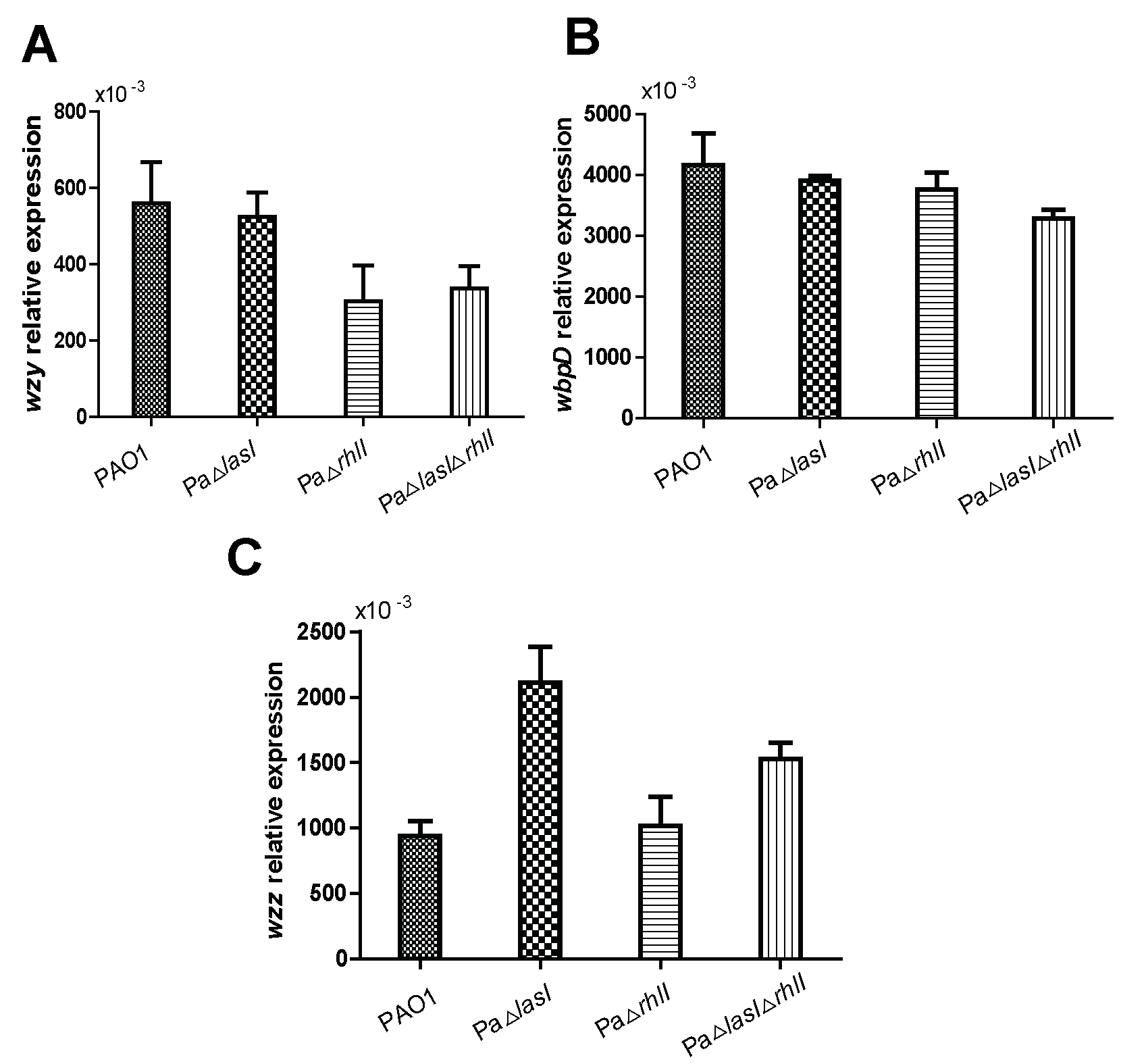

Supplement: FIG S4 [file mbio.03174-21-sf004.tif]
