# Supplementary material for: Quorum Sensing Promotes Phage Infection in Pseudomonas aeruginosa PAO1
Source: mBio. 2022 Jan 18;13(1):e03174-21. doi: 10.1128/mbio.03174-21 (PMC8764535; doi:10.1128/mbio.03174-21)
Supplement: TABLE S1 [file mbio.03174-21-st001.docx]

**Table S1 Strains and plasmids used in this study.**

| **Strain/plasmid** | **Characteristic** | **Source** |
| --- | --- | --- |
| ***Escherichia coli* strains** |  |  |
| DH5ɑ | Cloning strain | Invitrogen |
| BL21(DE3) | Cloning strain | Invitrogen |
| S17-1 | Transfer strain | Teng ^a^ |
| ***P. aeruginosa* strains** |  |  |
| PAO1 | Wild type | ATCC 15692 |
| PaΔ*lasI* | PAO1 mutant with *lasI* genes disrupted | This study |
| PaΔ*rhlI* | PAO1 mutant with *rhlI* genes disrupted | This study |
| PaΔ*lasI*Δ*rhlI* | PAO1 mutant with *lasI* and *rhlI* genes disrupted | This study |
| PΔ*lasI::lasI* | *lasI* complementary strain 1 | This study |
| PaΔ*lasI*Δ*rhlI::lasI* | *lasI* complementary strain 2 | This study |
| ***Plasmids*** |  |  |
| pK18mobsacBtet | \| Km^r^ and Tet^r^, *sacB*, RP4 oriT, ColE1 ori; suicide vector \| \| --- \| | This study |
| pBBR1mcs5 | Gm, broad host range | Kovach ^b^ |
| pBBR5-*lasI* | pBBR1mcs5 containing *lasI* | This study |

^a^ Teng F, Murray BE, Weinstock GM. 1998. Conjugal transfer of plasmid DNA from Escherichia coli to enterococci: a method to make insertion mutations. Plasmid 39:182–186. Doi:10.1006/plas. 1998.1336.

^b^ Kovach ME, Elzer PH, Hill DS, Robertson GT, Farris MA, Roop RM 2nd, Peterson KM. 1995. Four new derivatives of the broad-host-range cloning vector pBBR1MCS, carrying different antibiotic-resistance cassettes. Gene. 166:175-176.
