# Supplementary material for: Quorum Sensing Promotes Phage Infection in Pseudomonas aeruginosa PAO1
Source: mBio. 2022 Jan 18;13(1):e03174-21. doi: 10.1128/mbio.03174-21 (PMC8764535; doi:10.1128/mbio.03174-21)
Supplement: TABLE S2 [file mbio.03174-21-st002.docx]

**Table S2 Primers used in this study.**

| **Primers** | **Sequence (5'-3')** | **Usage** |
| --- | --- | --- |
| lasI-uF | GCTATGACATGATTACGAATTCGACCTTGGATTCTCGAAGAT | Flanking up region for *lasI* disruption |
| lasI-uR | GTCATGAAACTTTATCGAACTCTTCGCGCC |  |
| lasI-dF | GTTCGATAAAGTTTCATGACGGGGACCTGT | Flanking down region for *lasI* disruption |
| lasI-dR | CCCGGGTACCGAGCTCGAATTGTCGCCTATCTCGGTATCAG |  |
| lasI-del-1 | GTCCGGGTTCACCGAAATC | To verify *lasI* disruption |
| lasI-del-2 | GTTGTTCATCGAAGCGGTCT |  |
| rhlI-uF | GCTATGACATGATTACGAATTATTTTGCCGTATCGGCAAGG | Flanking up region for *rhlI* disruption |
| rhlI-uR | ATCGCGACCATTCCAGCGATTCAGAGAGCAAT |  |
| rhlI-dF | ATCGCTGGAATGGTCGCGATCAGCTTCCC | Flanking down region for *rhlI* disruption |
| rhlI-dR | CCCGGGTACCGAGCTCGAATTTGCTCGGCGATGTGCAGC |  |
| rhlI-del-1 | TCTTCCCCCTCATGTGTGTG | To verify *rhlI* disruption |
| rhlI-del-2 | TTGTAGTCGCCAGTGGTGG |  |
| qPCR-galU-F | AACAAGCCGCTGATCCAGTA | Primers for RT-qPCR |
| qPCR- galU-R | AGCTCGTAGCTGATGTCGAA |  |
| qPCR-rplS-F | ATACCGTGATCGTCCAGGTC | Primers for RT-qPCR |
| qPCR-rplS-R | GGCTGTAGGTCTGGAAGGTA |  |
| qPCR-wzy-F | TATTGTGCCTTCCACCCTGT | Primers for RT-qPCR |
| qPCR-wzy-R | GTCCTACCCTGACCCAAGAG |  |
| qPCR-wbpD-F | ATCGAGCGCAAGGATCAGTA | Primers for RT-qPCR |
| qPCR-wbpD-R | ATAGTCACGCCACAGACGAT |  |
| qPCR-wzz-F | CTCCGTCGTTGAGTGATGTG | Primers for RT-qPCR |
| qPCR-wzz-R | CGATGACGAGTTCCATCAGC |  |
| pBBR5-lasI-1 | CACAGGAAACAGCTATGACCATGATCGTACAAATTGGTCGGC | Primers for construction of pBBR5-*lasI* |
| pBBR5-lasI-2 | GACTCACTATAGGGCGAATTTCATGAAACCGCCAGTCG |  |
| pBBR5-1 | AATTCGCCCTATAGTGAGTCG |  |
| pBBR5-2 | GGTCATAGCTGTTTCCTGTGTG |  |
